# A pillared-layered copper(i) halide-based metal–organic framework exhibiting dual emission, and piezochromic and thermochromic properties with a large temperature-dependent emission red-shift†

**DOI:** 10.1039/c7ra11950j

**Published:** 2018-01-08

**Authors:** Bingjing Xin, Jianwei Sang, Yi Gao, Guanghua Li, Zhan Shi, Shouhua Feng

**Affiliations:** Jilin Institute of Chemical Technology Jilin 132022 P. R. China; State Key Laboratory of Inorganic Synthesis and Preparative Chemistry, College of Chemistry, Jilin University Changchun 130012 P. R. China

## Abstract

We report a new copper halide-based compound [Cu_6_I_6_Br_2_C_16_H_32_N_4_] (1) with a 3D 2-fold interpenetrated framework structure. Upon excitation at 290 nm and 350 nm, compound 1 shows dual emission at *ca.* 500 nm and *ca.* 530 nm. As the temperature decreased from 300 K down to 6 K, the luminescent properties of compound 1 show large red shifts of 120 nm and 72 nm, respectively.

## Abstract 2

A pillared-layered copper(i) halide-based metal–organic framework [Cu_6_I_6_Br_2_C_16_H_32_N_4_] exhibiting dual emission, and piezochromic and thermochromic properties with a large temperature-dependent emission red-shift is reported.

## Graphical abstract

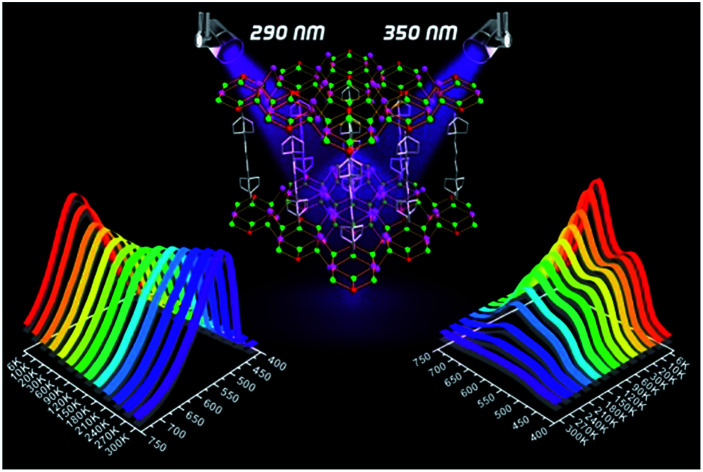

## Introduction

Copper(i) halide-based complexes have received much attention because of their excellent luminescent properties and various structures.^1^ Since the structure and luminescent properties of the copper(i) halide-based complex formulated [Cu_4_X_4_L_4_] (X = halogen, L = pyridine) were firstly reported by Hardt and his coworkers in the 1970s,^2^ a large number of copper halide compounds with interesting photoluminescent properties have been designed and prepared through various effective synthetic strategies.^3,4^ In recent years, several copper halides formulated as [Cu_4_I_4_L_4_] (L = P, S, N-based ligands) have been reported to show not only excellent luminescent properties but also response characteristics of the luminescence color to thermal or mechanical stimuli.^4,5^ Based on the results of theoretical computation and experimental research, the nature of the thermochromic luminescence or piezochromic luminescence of the copper halide complexes could be associated with two possible causes. One is halide to ligand charge transfer (XLCT) and the other is Cu–Cu interactions.^4*a*–*c*,6^ Generally, the blue shift is attributed to XLCT excited state and the red shift is attributed to cluster-centered transition.^5^ Although some studies about the thermochromic luminescence or piezochromic luminescence of the copper halide complexes have been performed, the direct evidence to prove that Cu⋯Cu interaction is responsible for thermochromic and piezochromic luminescence is still very limited.^4*a*,*b*,5*a*,7^ In this context, it is of significance to synthesize new copper halides with special luminescence behaviours and to clarify the emission mechanism. Herein, we report the synthesis, structure, and photoluminescence properties of a new copper halide-based compound [Cu_6_I_6_Br_2_L] (1) (L = C_16_H_32_N_4_ = 1,1′-(butane-1,4-diyl)bis(4-aza-1-azonia-bicyclo[2.2.2]octane)), which exhibits interesting luminescence behaviors, such as dual emission, thermochromic luminescence and piezochromic luminescence.

## Experimental

### Materials and methods

All reagents and solvents used were obtained from commercially available sources without further purification. Elemental analyses were performed on a Perkin-Elmer 2400 elemental analyzer. IR spectra were recorded within the 400–4000 cm^−1^ region on a Nicolet Impact 410 FTIR spectrometer using KBr pellets. Thermogravimetric experiments were performed with a TGA Q500 V20.10 Build 36 from room temperature to 800 °C at a heating rate of 10 °C min^−1^. X-ray photoelectron spectroscopy (XPS) data were collected on an ESCALAB 250 X-ray photo electron spectroscopy, using Mg Kα X-ray as the excitation source. X-ray powder diffraction (XRPD) patterns were taken on a Rigaku D/max 2550 X-ray powder diffractometer, with a speed of 1° min^−1^. The luminescence spectra were recorded on an Edinburgh Instruments FLS920 spectrofluorimeter from room temperature down to 6 K at a decreasing rate of 5 K min^−1^ (scan slit, 1.00 nm; fixed/offset slit, 1.00 nm; lamp, Xe900; step, 4.00 nm).

### Synthetic procedures

A mixture of CuBr_2_ (1.000 mmol, 0.223 g), HIO_4_·2H_2_O (1.000 mmol, 0.227 g), and NaClO_4_ (1.000 mmol, 0.141 g), C_6_H_12_N_2_·6H_2_O (1.000 mmol, 0.220 g), 1,4-dibromo butane (0.500 mL), H_2_O (5.000 mL) and HCl (65 μL, wt% = 0.36–0.38) was stirred for 24 h and then Na_2_CO_3_ (1.785 mmol, 0.150 g) was added into this solution and stirred for 12 h in air. This heterogeneous mixture was transferred and sealed in a 15 mL Teflon-lined steel autoclave. The autoclave was heated in an oven to 160 °C for 7 days. Then the autoclave was cooled over a period of 24 h to room temperature, and colorless block crystals were collected by filtration, washed with water and ethanol, and air dried (yield: 46% based on CuBr_2_). Anal. found: Cu, 20.42; C, 11.57; N, 4.55; H, 1.89. Calcd: Cu, 20.50; C, 11.62; N, 4.52; H, 1.95. IR: 3550 (m), 3447 (s), 3416 (s), 3236 (w), 3114 (m), 1638 (m), 1617 (s), 1457 (m), 1388 (s), 1316 (m), 1165 (w), 1047 (s), 995 (m), 843 (m), 787 (s), 606 (m), 478 (w).

### *In situ* reaction

**Scheme 1:**
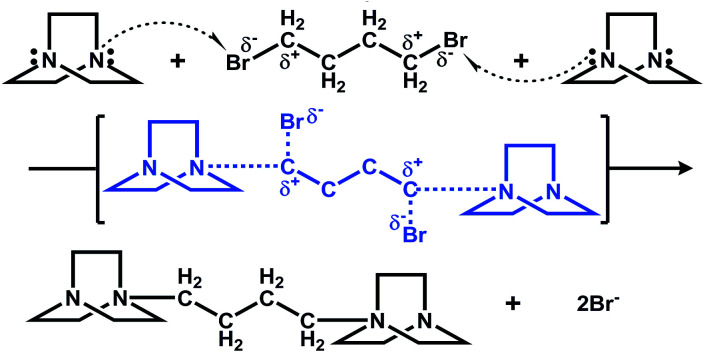
Mechanism of *in situ* reaction of DABCO and 1,4-dibromo butane in the synthesis of compound 1.

The results of structural analyses indicate that the structure of the ligands in the final products is different from that of the organic reagents. An *in situ* organic reaction occurred during the synthesis process according to the experiment results. In fact, several hydrothermal or solvothermal *in situ* organic reactions have been found in copper halide systems. In our study, the reagents 1,4-dibromo butane and DABCO react with each other to form a cationic ligand in the products, and the mechanism of this *in situ* alkylation is shown in Scheme 1.

## X-ray crystallography

### X-ray photoelectron spectroscopy

**Fig. 1:**
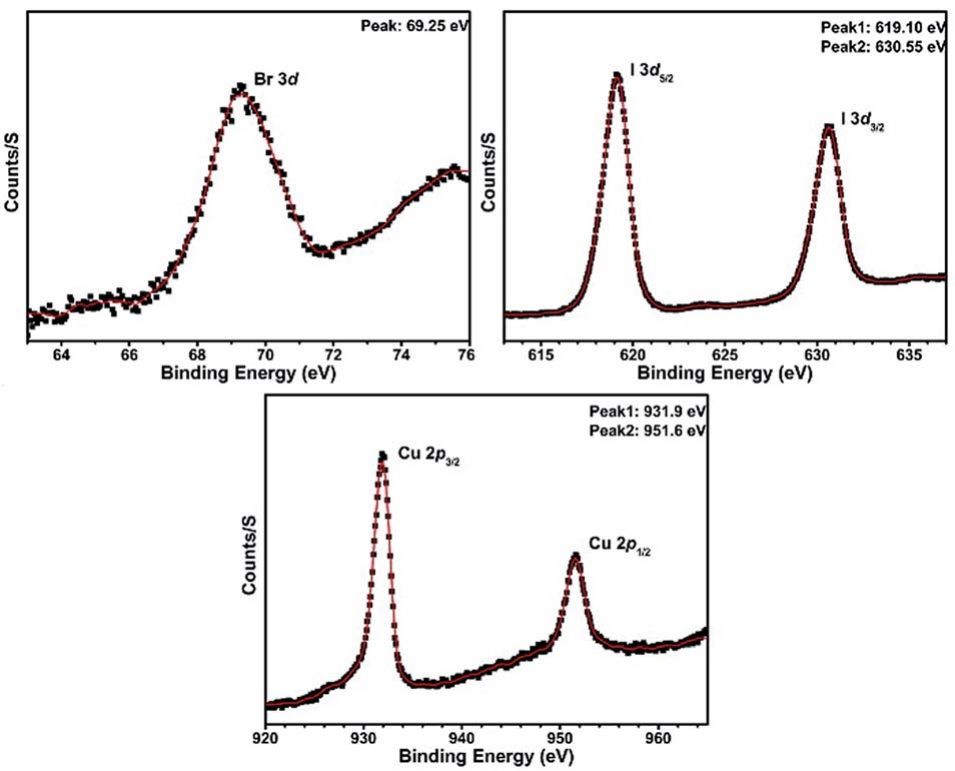
XPS spectra for compound 1.

X-ray photoelectron spectroscopy (XPS) experiments for compound 1 have demonstrated the existence of ions Cu^+^, I^−^ and Br^−^ in this compound (Fig. 1).

## Results and discussion

### Crystal structure

**Fig. 2:**
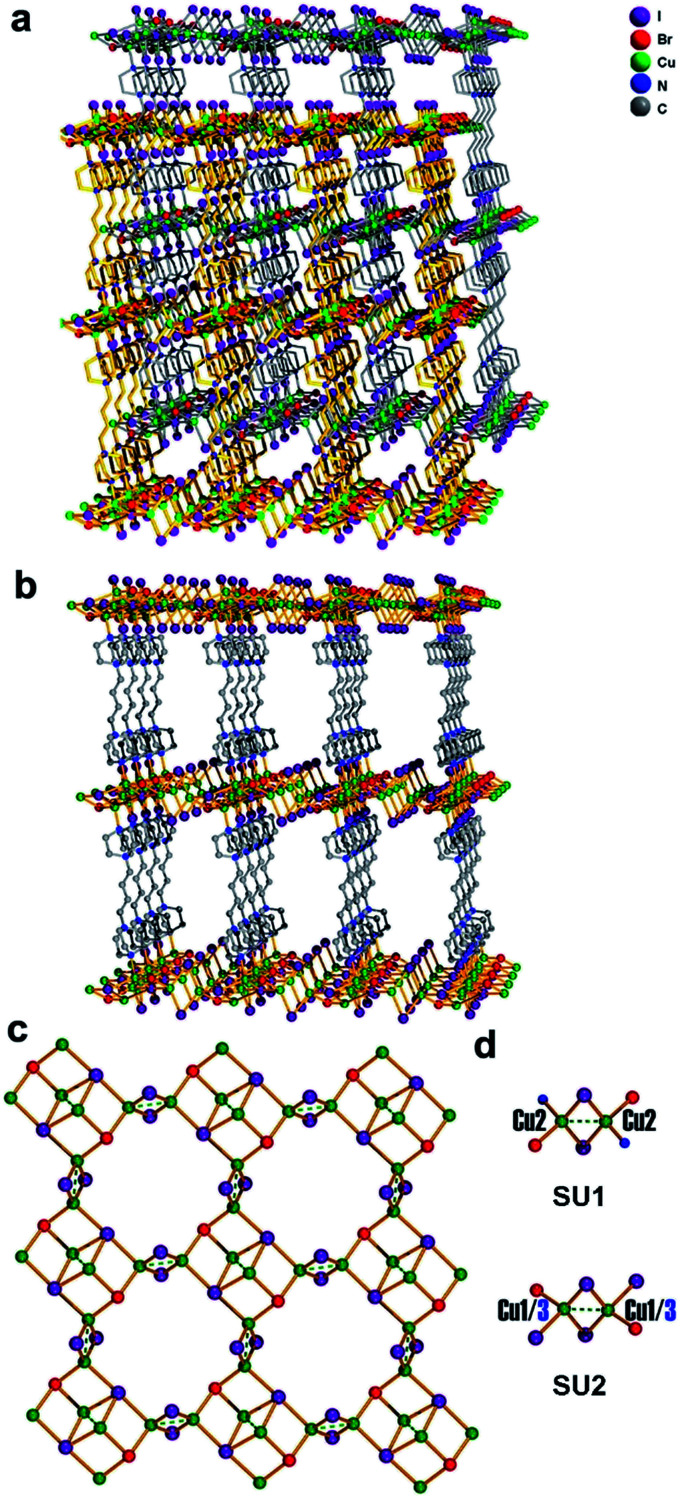
(a) 2-fold interpenetrated framework structure of compound 1. (b) Independent 3D framework pillared-layered structure of compound 1. (c) The structure of copper halide layer with the composition [Cu_6_I_6_Br_2_]^2−^. (d) The coordination environment of the Cu_2_I_2_ dimers [Cu_2_I_2_Br_2_N_2_] (SU1) and [Cu_2_I_4_Br_2_] (SU2).

Single crystal samples of compound 1 were obtained from hydrothermal reaction of CuBr_2_, HIO_4_, DABCO (1,4-diazabicyclo[2.2.2]octane), NaClO_4_, and 1,4-dibromo butane. Single crystal X-ray diffraction analysis reveals that compound 1 shows a 3D 2-fold interpenetrated framework structure (Fig. 2a). Each independent 3D framework is a pillared-layered structure and constructed with copper halide layer and cationic ligand L (L = 1,1′-(butane-1,4-diyl)bis(4-aza-1-azonia-bicyclo[2.2.2]octane)) (Fig. 2b). The cationic ligand L was obtained from *in situ* alkylation^8^ of the reagents 1,4-dibromo butane and DABCO (Scheme 1). The structure of each infinite copper halide layer with the composition [Cu_6_I_6_Br_2_]^2−^ is composed by rhombohedral Cu_2_I_2_ dimers through sharing μ_3_-Br and μ_4_-I (Fig. 2c). There are two types of coordination modes of the rhombohedral Cu_2_I_2_ dimers in the copper halide layer: [Cu_2_I_2_Br_2_N_2_] SU1 and [Cu_2_I_4_Br_2_] SU2 (Fig. 2d). The SU1 is coordinated with two bromine atoms and two nitrogen atoms from two different organic ligands and shows a *trans*-configuration. The SU2 is coordinated with two bromine atoms and two iodine atoms from two SU1 and adopts also a *trans*-configuration. The distance of Cu–I, Cu–Br, and Cu–N in the structure is 2.583(2)–2.883(2) Å, 2.5068(17)–2.542(2) Å, and 2.104(7) Å, respectively, which all falls into the normal range.^9^ In each dimer, there is an attractive interaction between two Cu centers approaching each other (Cu(1)–Cu(1) = 2.617(4) Å, Cu(2)–Cu(2) = 2.631(3) Å, Cu(3)–Cu(3) = 2.626(4) Å). On the other hand, judging from the distances of Cu–Cu (Cu(1)–Cu(2) = 3.202(9) Å, Cu(2)–Cu(3) = 3.211(7) Å), there is no interaction between the Cu centers belonging to different dimmers.

### Luminescence properties

**Fig. 3:**
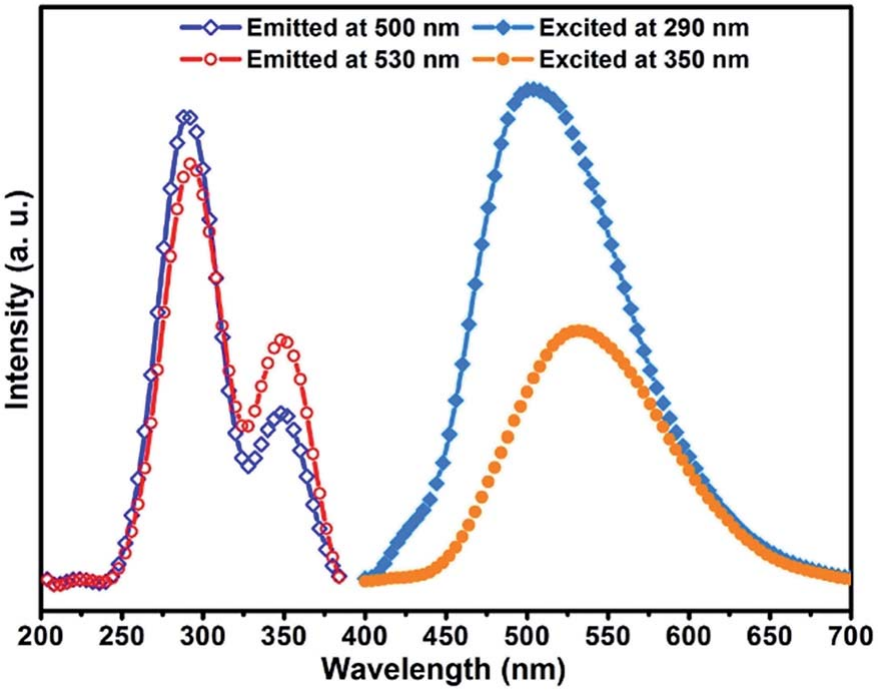
Excitation and emission spectra for compound 1 at room temperature.

The solid state powder samples of compound 1 show fascinating luminescence properties at ambient temperature. Upon excitation at 290 nm and 350 nm, the sample shows dual emission with a strong high energy (HE) emission band centred at *ca.* 500 nm and a weaker low energy (LE) emission band centred at *ca.* 530 nm (Fig. 3), respectively. On account of the ligands in the structure of compound 1 are saturated amine, the contribution of them to the luminescence should be ignored. Therefore, the luminescence emissions of compound 1 originated mainly from the contribution of the copper halide layers. Based on the reported literature, the emissions at room temperature should be mainly attributed to cluster-centred (CC*) transition with mixed halide-to-metal charge transfer (XMCT*) and metal cluster centred Cu^I^d^10^ → d^9^s^1^ transition (MCC*).^1*b*,10^ It is interesting to point out that most of the copper halide complexes reported so far exhibit single excitation-single emission characteristics, whereas our sample shows a dual emission characteristic. On the basis of the reported literature and our previous studies on the luminescence of copper halide complexes,^11^ we believe the dual emission behaviour of compound 1 should be attributed to the fact that there are two types of rhombohedral Cu_2_I_2_ dimers (SU1 and SU2) with different coordination environments in the structure of compound 1. Compared with the coordination nitrogen atom, the coordination iodine atom has larger electron density and gas-phase ionization energy which increase the electron-accepting character and lower the excited state energy level of rhombohedral Cu_2_I_2_ dimer. This will cause the excited energy of iodine coordinated dimer SU2 to be lower than that of nitrogen coordinated dimmer SU1.^12^ Recently, Li *et al.* reported a similar case, and they attributed the dual emission to two different copper cluster luminophores (Cu_4_I_4_ and Cu_3_Pz_3_).^13^ In order to clarify the luminescence mechanism of compound 1, the fluorescence emission and excitation spectra have been investigated at different wavelength. Firstly, monitored at different excitation wavelength from 240 nm to 390 nm, the emission band centred at 500 nm (*λ*_ex_ ≤ 290 nm) and 530 nm (*λ*_ex_ > 290 nm) (Fig. S1 in ESI†). The experiment results show that compound 1 has dual emission properties. Then, when emission wavelength from 400 nm to 680 nm was utilized to monitor the excitation spectra of compound 1, the results show that the excitation spectra both consist of two excitation bonds centred at 290 nm and 350 nm. However, the relative intensity radio of the excitation peak at 290 nm to the peak at 350 nm changed from 2.71 (monitored at 500 nm) down to 1.66 (monitored at 530 nm) (Fig. S2 in ESI†). Based on the aforementioned, it is easy to see that both of the two structure units SU1 and SU2 contribute to this dual emission phenomenon of the compound. Compared with SU2, which prefers to affect emission at 530 nm, SU1 predominantly contributes to emission at 500 nm.

**Fig. 4:**
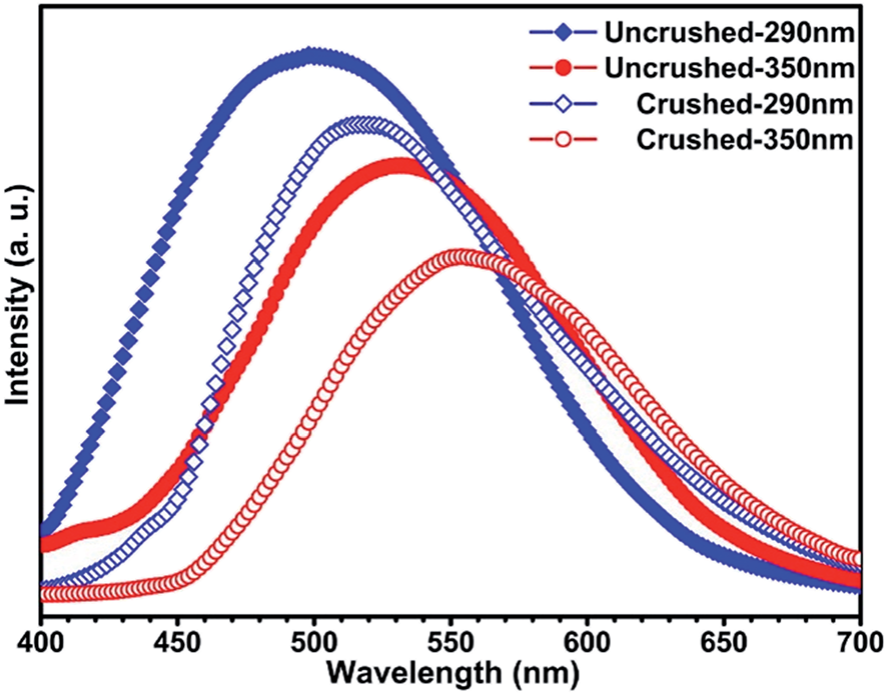
Emission spectra for compound 1 before and after being crushed upon excitation at 290 nm and 350 nm, respectively.

**Fig. 5:**
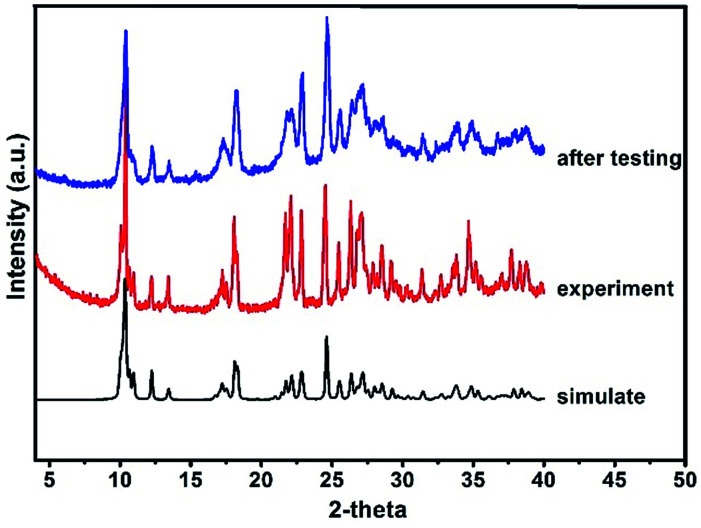
X-Ray powder diffraction patterns for compound 1.

Moreover, it is interesting to note that the luminescence properties of this compound are sensitive to pressure.^14^ When the solid state powder sample of compound 1 is pressed into a thick piece under 25 MPa pressure, its emission spectra are slightly red-shifted (Fig. 4). Excited at 290 nm, the emission peak of this compound is red shifted from 500 nm to 524 nm, and correspondingly from 530 nm to 556 nm upon excited at 350 nm. Excited at 290 nm and 350 nm, the corresponding red shift of the emission peak is about 25 nm, respectively. However, the XRD data of the compound before and after compressing are consistent (see Fig. 5), which means that the structure of the compound did not change after it was compressed. Such results indicate this red shift phenomenon might result from the weak bond contraction of the copper halide layer which is caused by pressing.

**Fig. 6:**
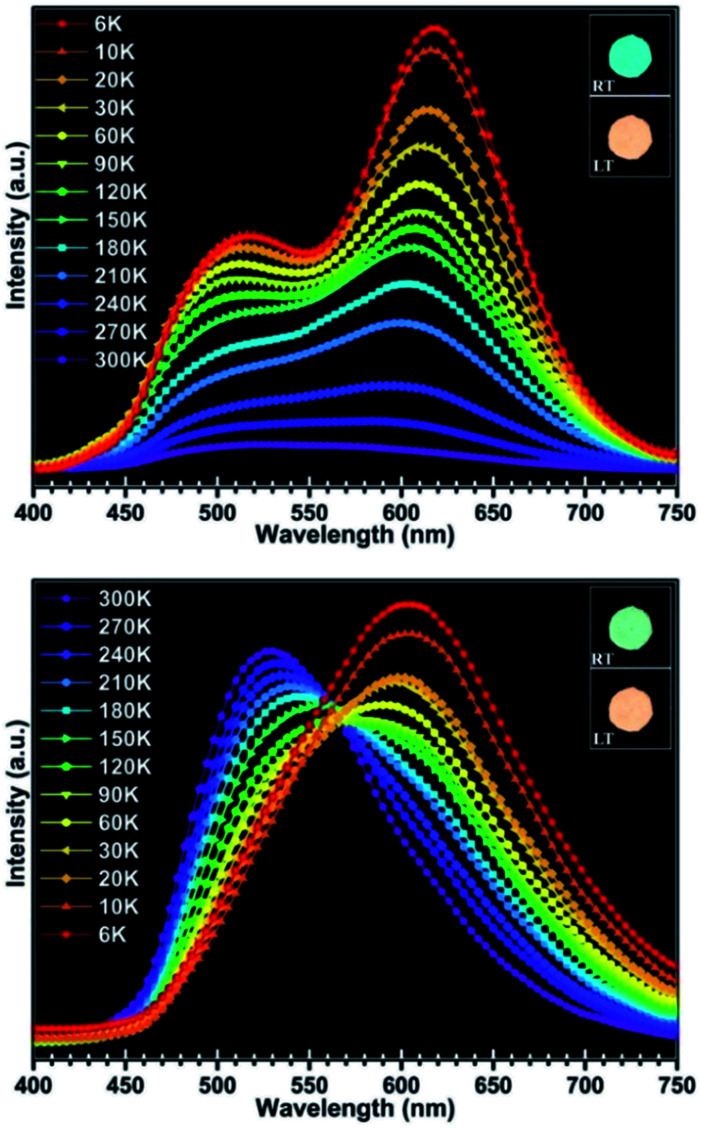
Solid-state luminescence spectra of compound 1 at 290 nm (up) and at 350 nm (down) between 300 K and 6 K (RT: room temperature; LT: lower temperature).

More interestingly, the luminescence properties of compound 1 are not only responsive to pressure, but also show a more sensitive response to temperature. Compound 1 exhibits luminescence with various colors at different temperature and the color change of luminescence under UV illumination can be easily observed by naked eyes (illustration in Fig. 6). Upon excitation at 290 nm, the maxima emission peak of compound 1 shifted from 500 nm to 620 nm as the temperature decreased from 300 K down to 6 K, and the red shift of the emission peak is about 120 nm (Fig. 6, Table 2). The luminescence colors at 300 K and 6 K can be quantified by the CIE (Commission International de L'Eclairage) 1931 chromaticity coordinates, and the values are *x* = 0.16, *y* = 0.32 and *x* = 0.47, *y* = 0.42, respectively. Under excitation at 350 nm, the luminescence of compound 1 showed similar change trend in the luminescence excited at 290 nm. The maxima emission peak in emission spectra shifted from 530 nm to 602 nm, and the shift value of the emission peak is about 72 nm (Fig. 6, Table 2). The CIE values of luminescence colors at 300 K and 6 K are *x* = 0.27, *y* = 0.46 and *x* = 0.45, *y* = 0.46, respectively. At present, several copper halide compounds with thermochromic luminescence properties have been reported,^15^ whereas in this work, the red-shift value is 120 nm. The orange luminescence of compound 1 could be easily reverted to blue-green and green, while the temperature changed back to 298 K. This indicates that compound 1 exhibits excellent reversibility during temperature detecting process.

To investigate the relationship between the luminescence behaviour and the crystal structure, we collected the crystal data for compound 1 at 293 K and 113 K (see Table 1). The crystal data reveal that no phase transition occurs in this compound with lowering the temperature. The main difference between the crystal structures is that bonds and angles show some variation at different temperature, wherein Cu–Cu distances in the structure become shorter with decreasing temperature (Table 3). Compared with Cu–Cu bond lengths, the other bond lengths such as Cu–X, Cu–N, C–C and C–N of compound 1 show smaller changes (see Table S1 in ESI†). Because the emission properties of compound 1 are related with halide-to-metal charge transfer (XMCT) and metal cluster centred Cu^I^d^10^ → d^9^s^1^ transition (MCC*) which originate from the copper halide layer, the luminescent changes of this compound should be attributable to the length changes of Cu–X and Cu–Cu. However, compared with the former one, the latter is more evident. Consequently, thermochromism luminescence of compound 1 is predominantly contributed to the cuprophilic interactions. When the temperature decreases, the bond length of Cu–Cu becomes shorter, leading to obvious bonding characteristics, which give rise to the lowering of the corresponding energy level. As a result, the emission spectra of this compound will be red-shifted. Simultaneously, the value of red shift excited at 290 nm is larger than that for 350 nm. This is because in structure units SU1 and SU2, the magnitudes of bond length change of Cu–Cu with decreasing temperature are different. According to the data shown in Table 3, the magnitude of changes in SU1 is larger than that in SU2. At room temperature, SU1 plays an important role for excitation at 290 nm and SU2 for 350 nm. Therefore, it could be revealed that the difference of red-shift value between excitation at 290 nm and 350 nm originated from the fact that the magnitude of changes for SU1 is larger than that for SU2.

**Table 1:** Crystal data and structure refinement for compound 1

Parameter	293 K	113 K
Formula	C_16_H_32_N_4_Cu_6_I_6_Br_2_	C_16_H_32_N_4_Cu_6_I_6_Br_2_
Formula weight	1582.92	1582.92
Crystal system	Triclinic	Triclinic
Space group	*P*1̄	*P*1̄
*a*, Å	9.856(2)	9.7150(19)
*b*, Å	9.951(2)	9.887(2)
*c*, Å	10.625(2)	10.541(2)
*α*, °	62.77(3)	62.33(3)
*β*, °	64.90(3)	66.13(3)
*γ*, °	84.22(3)	84.03(3)
*V*, Å^3^	833.4(3)	815.6(3)
*Z*	1	1
*D*_calc_, g cm^−3^	3.154	3.223
Goof	1.086	1.102
Final *R* indices [*I* > 2*σ*(*I*)]^a^^,^^b^	*R*_1_ = 0.0545, w*R*_2_ = 0.1433	*R*_1_ = 0.0463, w*R*_2_ = 0.1175
*R* indices (all data)	*R*_1_ = 0.0696, w*R*_2_ = 0.1528	*R*_1_ = 0.0529, w*R*_2_ = 0.1206

a *R*_1_ = ||*F*_o_| − |*F*_c_||/∑|*F*|_o_.

b w*R*_2_ = [∑w(*F*_o_^2^ − *F*_c_^2^)^2^/∑w(*F*_o_^2^)^2^]^1/2^, w = 1/[*σ*^2^(*F*_o_^2^) + (*ap*)^2^ + (*bp*)], *p* = [max(*F*_o_^2^, 0) + 2(*F*_c_^2^)]/3.

**Table 2:** Solid state emission for 1 was recorded from room temperature down to 6 K under excitation at 290 nm and 350 nm respectively

*λ*_ex_ (nm)	300 K	6 K	Shift (nm)
	*λ*_em_ (nm)	*λ*_em_ (nm)	
290	500	620	120
350	530	602	72

**Table 3:** Cu⋯Cu distances [Å] in compound 1 at two different temperatures

Bonds	293 K	113 K	*D*-value
Cu(1)⋯Cu(1)	2.617(4)	2.602(3)	0.015
Cu(2)⋯Cu(2)	2.631(3)	2.601(2)	0.030
Cu(3)⋯Cu(3)	2.626(4)	2.598(3)	0.028

## Conclusions

In summary, a new copper halide compound exhibiting interesting luminescence behaviours, such as dual emission, thermochromic luminescence and piezochromic luminescence, has been prepared through hydrothermal synthesis. In terms of the thermochromic luminescence properties, this compound displays a larger red-shift. Also, crystal structure data demonstrate that the temperature dependent variation of Cu⋯Cu distance is responsible for the luminescence thermochromism of copper halide compounds. Finally, this study provides strong data support for investigations of the temperature dependent property of this kind of compound.

## Conflicts of interest

There are no conflicts to declare.

## Supplementary Material

RA-008-C7RA11950J-s001

RA-008-C7RA11950J-s002

## References

[cit1] Armaroli N., Accorsi G., Cardinali F., Listorti A. (2007). Top. Curr. Chem..

[cit2] Hardt H. D., Gechnizdjani H. (1973). Z. Anorg. Allg. Chem..

[cit3] Peng R., Li M., Li D. (2010). Coord. Chem. Rev..

[cit4] Kim T. H., Shin Y. W., Jung J. H., Kim J. S., Kim J. (2008). Angew. Chem., Int. Ed..

[cit5] Perruchas S., Goff X. F. L., Maron S., Maurin I., Guillen F., Garcia A., Gacoin T., Boilot J. P. (2010). J. Am. Chem. Soc..

[cit6] Gong F., Wang Q., Chen J., Yang Z., Liu M., Li S., Yang G. (2010). Inorg. Chem..

[cit7] Cariati E., Bu X., Ford P. C. (2000). Chem. Mater..

[cit8] Li M., Li Z., Li D. (2008). Chem. Commun..

[cit9] Jin C. M., Zhu Z., Yao M. X., Meng X. G. (2010). CrystEngComm.

[cit10] Xue X., Wang X. S., Xiong R. G., You X. Z., Abrahams B. F., Che C. M., Ju H. X. (2002). Angew. Chem., Int. Ed..

[cit11] Xin B., Li Y., Zeng G., Peng Y., Li G., Shi Z., Feng S. (2012). Z. Anorg. Allg. Chem..

[cit12] Tsuge K., Chishina Y., Hashiguchi H., Sasaki Y., Kato M., Ishizaka S., Kitamura N. (2016). Coord. Chem. Rev..

[cit13] Zhan S. Z., Li M., Zhou X. P., Wang J. H., Yang J. R., Li D. (2011). Chem. Commun..

[cit14] Kunzelman J., Kinami M., Crenshaw B. R., Protasiewicz J. D., Weder C. (2008). Adv. Mater..

[cit15] Benito Q., Goff X. F. L., Maron S., Fargues A., Garia A., Martineau C., Taulelle F., Kahlal S., Gacion T., Boilot J. P., Perruchas S. (2014). J. Am. Chem. Soc..

