# Supplementary material for: A pillared-layered copper(i) halide-based metal–organic framework exhibiting dual emission, and piezochromic and thermochromic properties with a large temperature-dependent emission red-shift
Source: RSC Adv. 2018 Jan 8;8(4):1973–8. doi: 10.1039/c7ra11950j (PMC9077280; doi:10.1039/c7ra11950j)
Supplement: RA-008-C7RA11950J-s001 [file RA-008-C7RA11950J-s001.pdf]

Electronic Supplementary Material (ESI) for RSC Advances.
This journal is © The Royal Society of Chemistry 2017

Supporting Information

A pillared-layered copper (I) halide-based metal-organic framework exhibiting dual emission, piezochromisim, and thermochromisim properties with a large temperature-dependent emission red-shift

Table S1 The bond lengths of compound **1** at 293K and 113K. (Å)

Bond	293 K	113 K	Change
Cu(1)-I(1)	2.883(2)	2.825(2)	-0.058
Cu(1)-I(2)	2.631(2)	2.6395(19)	0.0084
Cu(1)-I(2)#2	2.593(2)	2.583(2)	-0.01
Cu(1)-Br(1)	2.542(2)	2.531(2)	-0.011
Cu(2)-I(1)	2.675(2)	2.6875(17)	0.0105
Cu(2)-I(1)#1	2.694(2)	2.6483(17)	-0.049
Cu(2)-Br(1)	2.5068(17)	2.5086(16)	0.0018
Cu(3)-I(1)	2.864(2)	2.857(2)	-0.007
Cu(3)-I(3)	2.583(2)	2.600(2)	0.017
Cu(3)-I(3)#3	2.644(2)	2.6260(18)	-0.018
Cu(3)-Br(1)#1	2.538(2)	2.535(2)	-0.003
N(1)-C(1)	1.475(13)	1.500(12)	0.025
N(1)-C(5)	1.507(12)	1.511(12)	0.004
N(1)-C(7)	1.511(11)	1.512(13)	0.001
N(1)-C(3)	1.523(13)	1.528(12)	0.005
N(2)-C(4)	1.473(15)	1.480(12)	0.007
N(2)-C(6)	1.479(12)	1.485(13)	0.006
N(2)-C(2)	1.488(15)	1.498(12)	0.010
C(1)-C(2)	1.524(15)	1.549(13)	0.025
C(3)-C(4)	1.531(15)	1.526(14)	-0.005
C(5)-C(6)	1.527(14)	1.518(13)	-0.009
C(7)-C(8)	1.532(14)	1.513(14)	-0.019
C(8)-C(8)#4	1.523(18)	1.528(19)	0.005

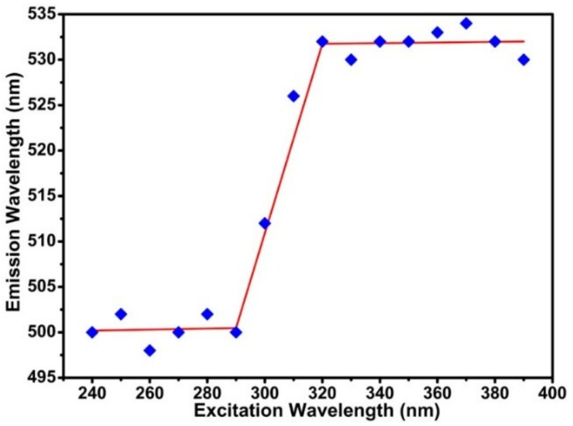

Figure S1 The emission wavelength of compound **1** upon excitation from 240 nm to 390 nm at room temperature.

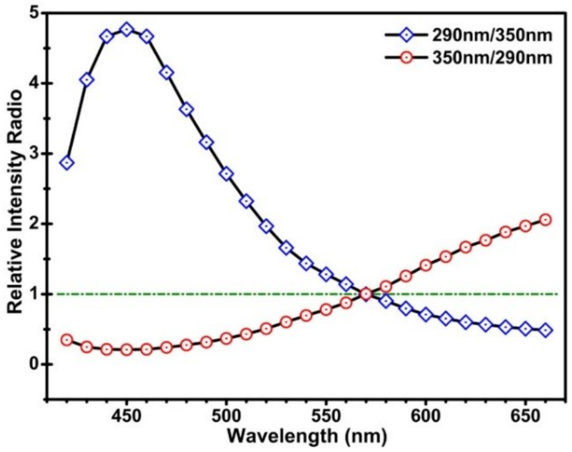

Figure S2 The relative intensity ratio of the excitation peak at 290 nm to the peak at 350 nm.

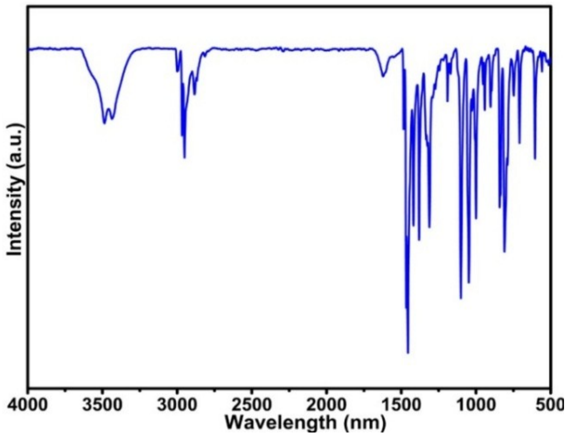

Figure S3 IR spectra of compound **1**.

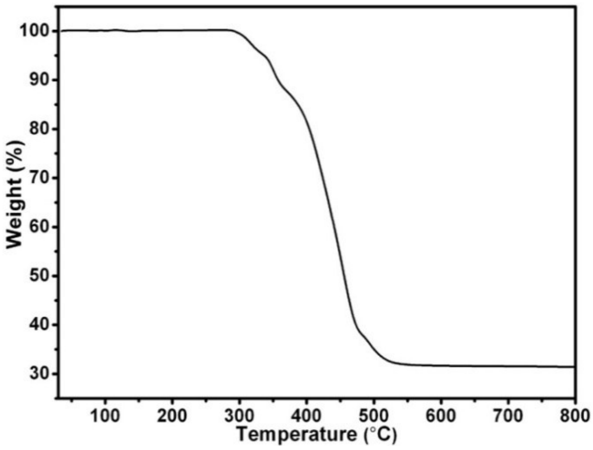

Figure S4 TGA curves for compound **1**.

Thermal behaviour studies of compound **1** were performed in air from room temperature to 800 °C at 10 °C /min. The TG curves for them show that compound **1** can be stable up to ca. 300 °C. In the temperature range 300-540 °C weight loss occurs due to the sublimation of iodine, bromine and organic ligand. Finally, the copper (I) ions of compound **1** react with oxygen and are converted to CuO, which is stable in air.

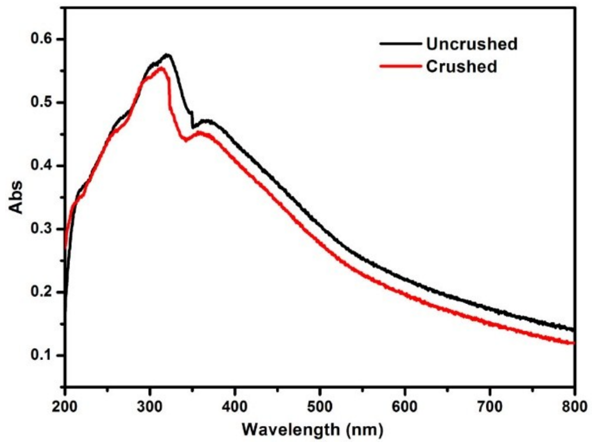

Figure S5 UV spectra of compound **1** before and after crushed.

In the spectra, the absorption strength of this compound slightly decreases after being crushed, and the adsorption of this compound is from 280 nm to 400 m.

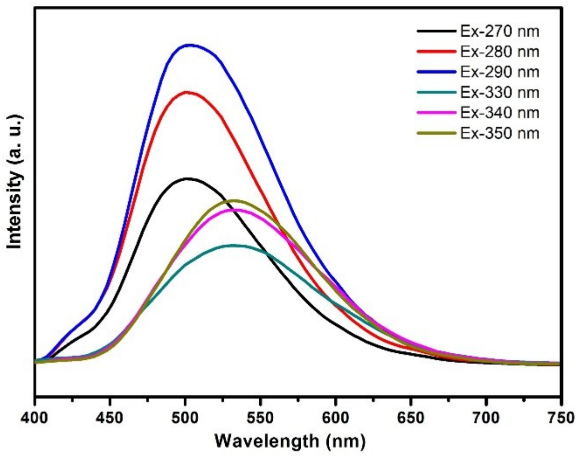

Figure S6 Emission spectra for compound **1** at room temperature. (Ex = 270 nm, 280nm, 290 nm, 330 nm, 340 nm and 350 nm)

According to the spectra, it's obvious to find that the emission peaks at 500 nm and 530 nm do not shift, respectively. As a consequence, it is safe to eliminate the possibility of Rayleigh scattering and Raman scattering from our emission spectra.
